# Finger-tracking captures distinct cognitive signatures in L1 vs. L2 reading

**DOI:** 10.3389/fpsyg.2026.1792437

**Published:** 2026-07-01

**Authors:** Eva Polo, Claudia Marzi

**Affiliations:** 1 Department of Philology, Literature and Linguistics, University of Pisa, Pisa, Italy; 2 Institute for Computational Linguistics, National Research Council, Pisa, Italy

**Keywords:** behavioral proxy of neural activity, finger-tracking, first and second language processing, linguistic violations, predictive processing, reading dynamics

## Abstract

**Introduction:**

Predictive and integrative mechanisms play a central role in real-time language comprehension. Understanding how native (L1) and non-native (L2) readers process linguistic information in real time is central to psycholinguistic research. While eye-tracking and event-related potentials (ERPs) have long provided insights into predictive and integrative mechanisms, behavioural methods with fine-grained temporal resolution remain comparatively limited. This study introduces a *finger-tracking* paradigm as a novel, fine-grained method for capturing real-time reading behaviour as participants trace sentences on a touchscreen.

**Methods:**

Two groups of young adult participants – native English speakers (L1) and upper-intermediate English L2 learners – read sentences containing either semantic or morphosyntactic violations, or well-formed control sentences. Tracking speed were analysed both at the token (whole word) and symbol (within-word position) levels.

**Results:**

L1 readers were overall faster than L2 readers and exhibited stronger sensitivity to linguistic anomalies, with modulation of tracking speed during violation processing. In contrast, L2 readers showed a reduced and more gradual sensitivity to violations, consistent with differences in the temporal dynamics of processing across the two groups.

**Discussion:**

In line with neurocognitive and eye-tracking evidence, these findings suggest that L1 reading is supported by rapidly deployed predictive mechanisms that are promptly disrupted by violations, whereas L2 reading is characterised by more incremental integration and reduced anticipatory processing. Overall, finger-tracking emerges as a sensitive, fine-grained behavioural method for studying real-time language processing, revealing distinct cognitive signatures in L1 and L2 reading.

## 1 Introduction

Reading is one of the most complex cognitive skills humans perform, involving the continuous integration of perceptual, lexical, syntactic, and semantic information over time. Far from being a passive decoding process, reading comprehension goes beyond simple word recognition, requiring readers to coordinate information across word, sentence, and text levels to build coherent mental representations. In doing so, multiple linguistic and cognitive resources must be dynamically engaged, including memory, attention, and predictive mechanisms that allow readers to anticipate upcoming linguistic input and adjust their expectations in real time. Recent theoretical frameworks have conceptualized language reading and comprehension as a probabilistic and predictive process, in which incoming words are continuously evaluated against internal models of linguistic structure (Kuperberg and Jaeger, 2016; Pickering and Garrod, 2013). These predictive operations make processing and comprehension efficient, but also render the process sensitive to violations of expected patterns—whether semantic or morphosyntactic. When the linguistic input deviates from prediction, the reader must rapidly revise her/his expectations, a process that has been widely documented in electrophysiological research. Event-related potentials (ERPs), which measure brain activity time-locked to specific linguistic events, have been instrumental in revealing the temporal dynamics of language comprehension. Distinct ERP components are associated with different stages of processing: for example, unexpected or incongruent words typically elicit an enhanced N400 component, reflecting increased semantic integration costs (Li et al., 2024; Kuperberg and Jaeger, 2016; Kutas and Federmeier, 2011), whereas morphosyntactic anomalies often evoke a P600 response, linked to structural reanalysis or repair processes (Aurnhammer et al., 2021; Hagoort and Brown, 2000; Osterhout, 1997; Hagoort et al., 1993). Together, this body of evidence suggests that language reading and comprehension involves continuous cycles of prediction, verification, and updating.

Importantly, the timing and amplitude of the neural responses reveal not only whether a violation is detected, but also how the reader processes linguistic information—anticipatorily or reactively. Readers who rely strongly on predictive processing tend to show early and robust ERP responses to violations, whereas those adopting a more bottom-up, integrative strategy exhibit delayed or attenuated effects (Pickering and Gambi, 2018; Van Petten and Luka, 2012). Understanding this balance between predictive and reactive processing therefore offers a powerful window into the dynamics of language processing and comprehension.

Traditional experimental paradigms such as ERPs and eye-tracking have provided compelling evidence for the temporal and hierarchical organization of language processing. Beside the N400 and P600 components as well-established neural markers of lexical-semantic and syntactic prediction errors (Seyednozadi et al., 2021; Kutas and Federmeier, 2011), eye-tracking studies have, in turn, shown that fixation durations increase when readers encounter words that violate syntactic or semantic expectations, reflecting the cognitive cost of integration and reanalysis (Hubers et al., 2020; Staub, 2015).

Importantly, predictive mechanisms in reading are not uniform across readers but vary as a function of linguistic experience, proficiency, and processing efficiency. Differences in how readers generate, maintain, and update predictions offer a crucial testing ground for theories of real-time language comprehension. In particular, populations that differ in the strength and automaticity of their linguistic representations provide a unique opportunity to examine how predictive and reactive mechanisms are weighted during reading. Second language (L2) readers constitute a paradigmatic case in this respect. Compared to native (L1) readers, they typically operate under increased cognitive demands and reduced processing efficiency, which may affect both the timing and the robustness of prediction-based mechanisms.

### 1.1 Predictive and reactive mechanisms in L1 and L2 reading

A growing body of research suggests that predictive processing mechanisms differ markedly between native (L1) and non-native (L2) speakers. In L1 comprehension, predictions about upcoming words are generated rapidly and automatically, supported by well-entrenched linguistic representations and probabilistic expectations derived from extensive language exposure. In contrast, L2 readers—particularly late or less proficient learners—tend to rely more heavily on local cues and bottom-up information (Kaan, 2014; Hahne, 2001). These differences reflect not only linguistic knowledge but also differences in processing efficiency and resource allocation (Schlenter, 2023; Kuperberg and Jaeger, 2016).

Neurocognitive evidence has consistently shown that L2 readers exhibit attenuated and delayed neural markers of prediction. Event-related potential (ERP) studies report reduced and temporally shifted N400 and P600 components in L2 comprehension, suggesting that prediction and integration occur later or less efficiently (see, for reviews, Kuperberg and Jaeger, 2016; Van Hell and Tokowicz, 2010). It has been shown that L2 learners tend to be less sensitive than L1 speakers to the conflict between syntactic and semantic processing of a same stimulus (Zheng and Lemhöfer, 2019). Similarly, Hopp (2015a) and Kaan (2014) argue that reduced automaticity in lexical and syntactic access constrains L2 readers' ability to pre-activate upcoming information. Consequently, L2 processing often appears more reactive—that is, based on integration after the input is encountered—whereas L1 processing is predominantly predictive, anticipating incoming words before they appear.

Behaviorally, this distinction is reflected in longer and more variable reading times, greater sensitivity to lexical and morphosyntactic complexity, and reduced contextual facilitation effects in L2 compared to L1 readers (Cop et al., 2015; Kuiken and Vedder, 2012; Kotz, 2009). Eye-tracking studies show that L2 readers often integrate information later, with delayed regressions and weaker parafoveal preview effects (Tiffin-Richards, 2024; Nahatame, 2023).

However, while electrophysiological and eye-movement studies have provided compelling evidence for differences between L1 and L2 readers in the timing and strength of predictive mechanisms, relatively few behavioral paradigms can capture these dynamics online during continuous, ecologically valid reading. This limitation is particularly relevant when the goal is to characterize how prediction and integration unfold moment-by-moment under different processing constraints.

### 1.2 Finger-tracking as a behavioral window into reading dynamics

*Finger-tracking* offers a simple yet powerful way to capture the continuous temporal unfolding of reading behavior. By requiring readers to trace the text as they read, this paradigm produces a fine-grained, time-resolved behavioral signal that closely follows the progression of linguistic input across words and within words. Unlike eye movements, which primarily index shifts of overt visual attention and segment reading into discrete fixation events, finger movements provide a continuous motor trace that unfolds alongside ongoing linguistic processing. At the same time, finger-tracking does not impose the physical constraints associated with electrophysiological recordings and can be implemented in relatively naturalistic reading conditions using the touchscreen of an ordinary tablet. The resulting continuous movement trajectory provides a high-resolution record of reading time. Finger-tracking does not require specialized hardware or calibration, making it suitable for classroom or field studies, while still offering precise temporal and spatial information. As such, it provides a low-cost and ecologically valid behavioral measure that complements traditional eye-tracking approaches, and offers new insights into how visual and motor processes are coordinated during text reading.

In detail, participants continuously trace linguistic stimuli presented on a touchscreen, producing a rich stream of kinematic data that reflects the unfolding of lexical access. Previous research on continuous movement measures has shown that movement trajectories can provide a sensitive behavioral index of ongoing cognitive processes, reflecting the temporal dynamics of information processing as cognition unfolds over time (Freeman et al., 2011; Song and Nakayama, 2009). Accordingly, subtle variations in tracking speed and timing can reveal the cost of prediction, integration, and error detection as the linguistic input unfolds. In this sense, finger-tracking captures not only *when* readers slow down, but also *where* within the word or sentence processing difficulty emerges, offering a spatially and temporally continuous view of online processing.

Finger-tracking has recently been applied across a variety of reading populations, providing converging evidence for its sensitivity to the temporal dynamics of reading. Studies in both adult and developing readers have shown that finger-tracking measures strongly correlate with established eye-tracking indices, thereby supporting its use as a behavioral proxy for the temporal dynamics of oculomotor control and lexical processing (Marzi et al., 2025; Nadalini et al., 2023; Crepaldi et al., 2022). Similarly, in clinical populations, including individuals with mild cognitive impairment and early dementia, finger-tracking has proven effective in detecting subtle alterations in online comprehension and reading strategies (Marzi et al., 2026). Together, these evidence support the ecological validity and generalizability of the finger-tracking paradigm as a fine-grained method for investigating reading dynamics.

Here, we apply finger-tracking to investigate how native and non-native readers differ in their real-time processing of linguistic violations. While native readers are typically thought to rely on highly predictive, top-down mechanisms (Pickering and Clark, 2014), L2 learners often show more reactive and locally driven processing patterns (Kaan, 2014). By examining the temporal signatures of finger movement at both the word (token) and within-word (symbol) levels, we aim to capture how predictive and integrative processes interact across different grain sizes of linguistic input during reading. We hypothesize that L1 readers will show earlier and more pronounced sensitivity to linguistic violations, reflecting disruption of anticipatory processing, whereas L2 readers may exhibit reduced or more gradual effects, consistent with differences in the temporal dynamics of predictive processing.

### 1.3 The present study

Building on these premises, the present study investigates whether finger-tracking can capture distinct real-time reading dynamics in native (L1) and non-native (L2) English speakers. Participants read English stimuli containing either semantic or morphosyntactic violations, along with matched correct control items. This experimental design enables a direct comparison of reading behaviors at two complementary levels of analysis: the token level (whole words) and the symbol level (within-word positions).

At the whole-word level, we predicted longer tracking times for violation stimuli, reflecting increased processing demands and disruption on predictive flow. At the within-word level, we expected qualitatively distinct temporal profiles for L1 and L2 readers. For L1 speakers, sensitivity to violations should emerge early and consistently across word positions, consistent with predictive processing and rapid anomaly detection. For L2 speakers, by contrast, differences between correct and violation sentences were expected to emerge later and less distinctly within words, reflecting delayed or more locally driven integration processes.

This approach tests whether finger-tracking can serve as a behavioral proxy for the temporal dynamics of predictive vs. reactive processing, complementing neural evidence from ERP research. More broadly, it explores whether fine-grained temporal signatures of reading, captured through an ecological and accessible motor measure, can reveal cognitive differences in processing and comprehension between L1 and L2 readers.

If successful, this method could offer a bridge between controlled laboratory paradigms and naturalistic reading environments. It would also contribute to ongoing discussions on how prediction, automaticity, and proficiency shape the temporal architecture of comprehension in first and second language processing.

## 2 Materials and methods

### 2.1 Participants

Thirty-two young adults participated in the study, divided into two groups of sixteen each. The L1 group consisted of 16 native speakers of English, all residing and tested in Oxford (UK), while the L2 group included 16 English learners, whose first language was Italian, tested in Pisa (Italy). L2 proficiency was self-reported and corresponded on average to CEFR upper-intermediate (B2) level, based on at least 5 years of formal English instruction at secondary-school level, as well as ongoing use of English as a language of university study or in course materials. All participants reported normal or corrected-to-normal vision and no history of reading or language disorders.

Participants were enrolled at different educational levels, ranging from the final year of high school to postgraduate studies (12 − 16 years of formal education, mean = 14.02, standard deviation = 1.61). The two groups were comparable in terms of age (in the range 18–35, mean age 25.7 for L1 and 25.9 for L2) and educational background (mean years of formal schooling 13.9 for L1 and 14.2 for L2). Specifically, the sample included 12 high school students (7 L1, 5 L2), 15 bachelor students (6 L1, 9 L2), and 5 master's students (3 L1, 2 L2).

All participants took part voluntarily and provided written informed consent prior to participation. The study adhered to the ethical standards of the previously approved protocol on developmental reading (Ethical approval 0037523/2021, Italian National Research Council—Committee for Research Ethics) and complied with the principles of the Declaration of Helsinki.

### 2.2 Materials

The experimental materials consisted of 16 English sentences, each constructed in two versions: a correct version and a violated version. Violations were of two types—semantic and morphosyntactic—yielding a total of 32 stimuli. Semantic violations involved contextually anomalous words (e.g., The volcano was meticulously *eaten by a team of experienced scientists using advanced thermal imaging equipment, correct version: *observed*), while morphosyntactic violations introduced ungrammatical agreement or tense errors (e.g., The old house *has broken into while the owners were on vacation in the mountains, correct version: *was*). These types of manipulations are commonly used in psycholinguistic research to elicit sensitivity to semantic implausibility and morphosyntactic violations, typically producing robust processing costs in both behavioral and neurocognitive measures (e.g., Osterhout 2015; Hagoort 2003; Kutas and Hillyard 1983).

All sentences in their correct versions were designed to be comprehensible for upper-intermediate L2 readers, and lexical selection was controlled to avoid low-frequency or highly specialized lexical items. Many stimuli consisted of two or more clauses, typically separated by a comma or a full stop. Overall, stimuli were relatively long, with an average of 25.56 tokens per sentence (sd = 5.0, range = 17–35) and a mean token length of 4.50 characters (sd = 0.76, range = 3.6–6.1), ensuring that the reading task required sustained processing across multiple words. Each participant was presented with 16 stimuli: eight correct and eight containing a violation. Stimulus assignment was counterbalanced across participants, such that each stimulus appeared equally often in its correct and violated form across the two groups. This ensured that no participant saw both versions of the same item.

Since the experimental design aimed to approximate relatively naturalistic reading conditions while maintaining control over the occurrence of semantic and morphosyntactic violations, no filler items were included, as the study prioritized continuity of online reading dynamics rather than to function as a classical acceptability-judgement paradigm. Whereas filler items are commonly used in highly controlled psycholinguistic paradigms to reduce strategic responding (see, for a recent review, Covey and Gabriele, 2023), the inclusion of well-formed control items introduced variability in sentence acceptability and reduced the predictability of anomalous trials. Additionally, recent evidence suggests that filler composition itself may shape participants' expectations and processing behavior (Arehalli and Wittenberg, 2021). This consideration is particularly relevant in paradigms investigating predictive processing, where the statistical structure of the experimental environment may directly influence anticipatory behavior. Each stimulus was followed by either a comprehension question or a grammaticality judgement. This combination served to maintain attention throughout the task and encouraged participants to engage both with sentence meaning and with linguistic well-formedness during continuous reading.

Each stimulus was presented in a sans-serif font (Arial, 21.25 pt) on a 10.1-inch Samsung tablet, with a 14.9 cm × 24.5 cm touchscreen at a resolution of 1,920 × 1,200 pixels. This setup allowed participants to trace each sentence with their finger while reading, providing a fine-grained, temporally precise measure of reading behavior.

### 2.3 Procedures

Participants were tested individually in a quiet room to minimize external distractions. After a brief familiarization phase consisting of a practice trial, they were instructed to read each stimulus silently while tracing the text with their index finger on the touchscreen. They were encouraged to move their finger smoothly and at their natural reading pace.

After reading each stimulus, a comprehension question appeared on screen, requiring a yes/no response (e.g., “Is this sentence correct?” or “Did the agent x make the action?”). Responses were given by tapping on the corresponding option, and both response accuracy and reaction times were recorded. These questions were included to ensure attentive reading but were not the primary dependent measure of our analyzes.

The entire experiment lasted approximately 30 min per participant, including instructions and the main reading task.

### 2.4 Data acquisition and alignment

Reading sessions were conducted on a tablet in landscape orientation. The *Readlet* application (Marzi et al., 2025; Nadalini et al., 2023; Taxitari et al., 2021; Marzi et al., 2020; Ferro et al., 2018) recorded finger movements directly via the tablet's touchscreen interface. Finger trajectories are captured with an effective sampling rate ranging from approximately 60–120 Hz, producing a continuous stream of spatially and temporally resolved touch coordinates, each encoded with its position on the screen and its timestamp. This allowed reconstruction of raw finger movement trajectories during reading. Text-to-finger alignment was performed using a convolution-based mapping procedure that identified the optimal correspondence between continuous touch-event sequences and textual layout. For each uninterrupted sequence of touch events within a letter-specific spatial region, tracking time was computed as the temporal difference between the first and last event. Letter-level tracking times were then aggregated into higher-level linguistic units (e.g., word tokens) by summation across constituent letters, yielding unit-level measures aligned with the stimulus structure.

This alignment procedure enabled a fine-grained reconstruction of the temporal evolution of finger position relative to the linguistic input, closely paralleling how a sequence of eye fixations is mapped onto a sequence of words (Crepaldi et al., 2022; Nadalini et al., 2023). This enables a fine-grained reconstruction of where the reader's finger was directed at any given point during reading and, indirectly, the locus of ongoing visual-motor engagement with the text.

### 2.5 Data analysis

Finger-tracking data were processed to derive two complementary measures at two levels of granularity: (i) the token level, corresponding to whole words, and (ii) the symbol level, corresponding to within-word positions.

At the token-level, total tracing time was computed as the duration between the first and last touch event associated with each given token. This measure provides a coarse-grained estimate of reading and lexical processing time.

At the symbol-level, finger trajectories were further resampled to produce fine-grained time series aligned with each orthographic character within a word. This measure captures subtle, within-word variations in finger movement dynamics that may reflect moment-to-moment processing difficulty.

The combination of token- and symbol-level analyses provides insight into how linguistic violations are detected and integrated over time in first and second language processing.

All statistical analyses and data visualizations were performed using R (version R-4.5.1, R Core Team, 2025).

Before statistical analyses, finger-tracking data were preprocessed to remove extreme outliers. Extreme values were trimmed using an empirically motivated cutoff of 1.3 seconds per token. This value lies well beyond the 99^th^ percentile of the distribution and isolates the long-tail observations associated with pauses or accidental touches. This procedure removed 0.97% of data points.

Trials with a tracking time of 0 milliseconds (indicating no touch event) were inspected separately prior to exclusion, to check for possible systematic differences across groups or sentence positions. The overall proportion of zero-duration events did not differ significantly between L1 and L2 readers (*Fisher*'s exact test, *p* = 0.62). However, their distribution across sentence positions did. A non-parametric *Kolmogorov-Smirnov* test comparing the positional distributions of zero-duration events revealed a significant difference between groups when position was expressed relative to the sentence end (*D* = 0.18, *p* = 0.002). Specifically, L1 readers showed a marked concentration of zero-duration events near sentence-final positions, consistent with anticipatory processing of the final tokens, whereas L2 readers displayed a more uniform distribution across positions. On the basis of this preliminary inspection, zero-duration events were excluded from subsequent analyses, corresponding to 2.90% of data points removed during preprocessing.

## 3 Results

### 3.1 Overall reading performance

Preliminary results indicated significantly longer tracking times for L2 than L1 readers overall, together with lower comprehension accuracy. A non-parametric *Kruskal–Wallis* test on tracking time by tokens confirmed a significant main effect for group [χ^2^(1) = 98.69, *p* < 2.2*e*−16], with longer times for L2 (mean = 0.27, median = 0.20) than for L1 readers (mean = 0.22, median = 0.17). Similarly, accuracy on comprehension and grammaticality questions differed significantly between groups [χ^2^(1) = 5094.8, *p* < 2.2*e*−16], with L2 readers showing lower participant-level proportion of correct responses (mean = 0.54, median = 0.55) compared to L1 readers (mean = 0.75, median = 0.82).

To evaluate whether finger-tracking captures well-established lexical determinants of reading time, we examined the influence of word length and frequency on token-level tracking times using *Generalized Additive Models* (GAMs). We firstly fitted a GAM with group (L1 vs. L2), word length (expressed as number of orthographic letters), and their interaction as fixed effects, and random intercepts for participants. Crucially, a significant group × length interaction emerged (*p* < 2*e*−16), with L2 readers showing a steeper increase in tracking time with word length compared to L1 readers. This pattern suggests that non-native readers can be more sensitive to surface-level orthographic demands as compared with native ones. The model explained approximately 50 % of the variance (adjusted R^2^ = 0.498). Full model specifications are provided in Table A1 in Appendix.

Next, we examined the influence of token frequency (log-transformed and derived from the *Subtlex* corpus, Van Heuven et al. 2014) on finger-tracking times fitting a GAM with the interaction of group and frequency, and random intercepts for participants. Results confirmed a strong facilitatory effect of frequency. L2 readers showed overall longer tracking times than L1 readers (*p* < 0.001), with a significantly stronger effect of frequency for L2 than for L1 (*p* < 8*e*−14), suggesting that L2 readers may depend more heavily on word-level statistical information during reading. The model explained approximately 41 % of the variance (adjusted R^2^ = 0.406). See Table A2 in Appendix for full model specifications.

Together, these preliminary results confirm that finger-tracking reliably captures canonical lexical influences on reading behavior, while also revealing systematic group differences in the strength of such effects.

**Figure 1:**
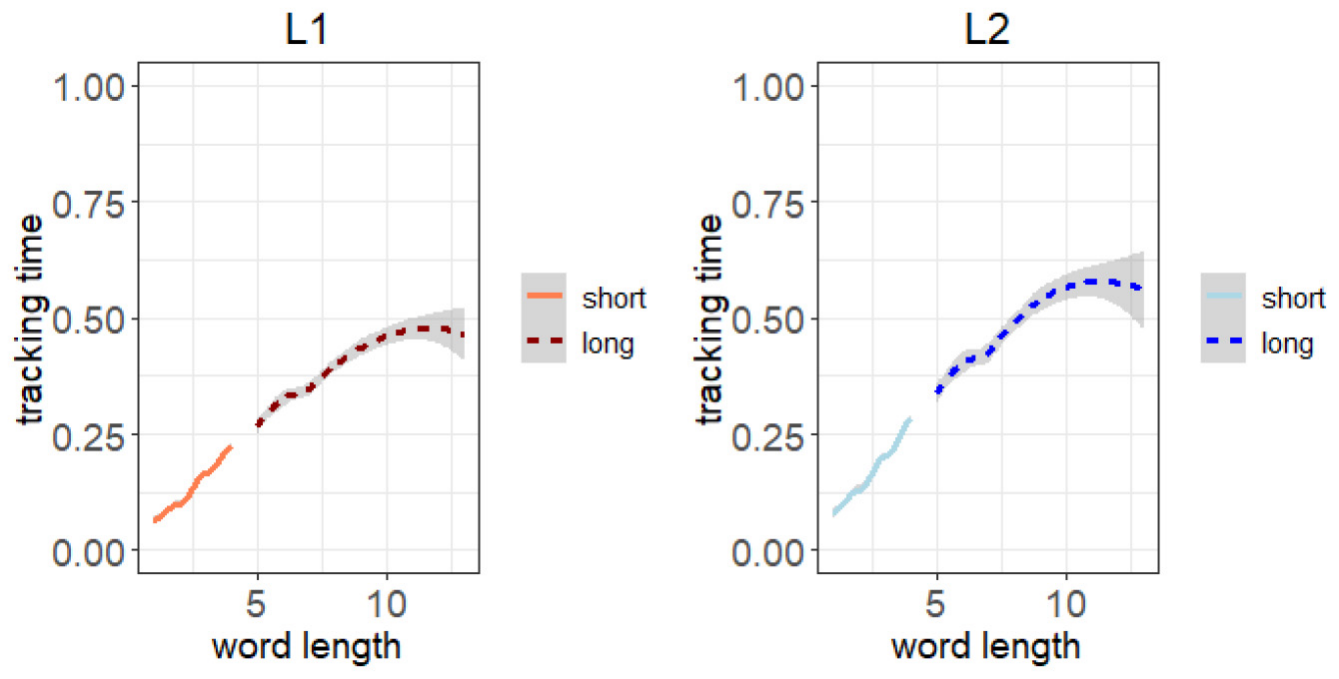
Non-linear regression plots (*ggplot* function, *loess* method) fitting tracking time in seconds for short ( ≤ 4 letters) and long (>4 letters) words, plotted separately for L1 and L2 readers. Shaded areas indicate 95% confidence intervals.

To further explore the effect of word length, tokens were divided into two groups: short ( ≤ 4 characters) and long (>4 ≤ 13 characters). Tracking times in seconds were analyzed fitting a GAM with fixed effects for group (L1 vs. L2), length group (short vs. long), their interaction, and a random intercept for participants. The model revealed a significant main effect of length group (*p* < 2*e*−16), with longer words associated with increased tracking times. While the baseline tracking time for short words did not differ significantly between L1 and L2 readers (*p* >0.05), the group × length interaction was highly significant (*p* < 7*e*−16), indicating that L2 readers show a steeper increase in tracking time for long words compared to L1 readers. Full model specifications are provided in Table A3 in Appendix. Figure 1 shows mean tracking times separately for L1 and L2 readers. The plot highlights that both groups exhibit longer tracking times for long words, but the effect is clearly amplified in L2 readers, suggesting greater surface-level orthographic demands.

Overall, these evidence suggest that L2 readers are slower and more sensitive to word-level orthographic properties, consistent with a more effortful, locally driven reading strategy.

### 3.2 Sensitivity to linguistic violations

Within-group comparisons revealed that tracking times were modulated by sentence well-formedness (correct vs. violation) in L1, but less so in L2. Specifically, for L1 readers, stimuli containing linguistic violations elicited significantly longer tracking times across all tokens compared to well-formed control stimuli [*Kruskal-Wallis* χ^2^(1) = 5.68, *p* = 0.017]. In contrast, no effect of sentence well-formedness was observed for L2 readers, whose tracking times did not significantly differ between violated and correct stimuli [*Kruskal–Wallis* χ^2^(1) = 0.02, *p* >0.05].

A parallel within-group analysis was conducted on response accuracy. For L1 readers, sentence well-formedness significantly affected overall accuracy, with lower accuracy for sentences containing violations compared to correct sentences [*Kruskal–Wallis* χ^2^(1) = 7.16, *p* < 0.01]. For L2 readers, the effect of sentence well-formedness did not reach conventional levels of statistical significance [*Kruskal–Wallis* χ^2^(1) = 3.63, *p* = 0.057], indicating only a marginal trend toward reduced accuracy for violated sentences. Thus, response accuracy mirrored the pattern observed in tracking times, with clear sensitivity to violations in L1 readers and a reduced or absent effect in the L2 group, in line with the overall lower accuracy previously observed in L2 readers.

**Figure 2:**
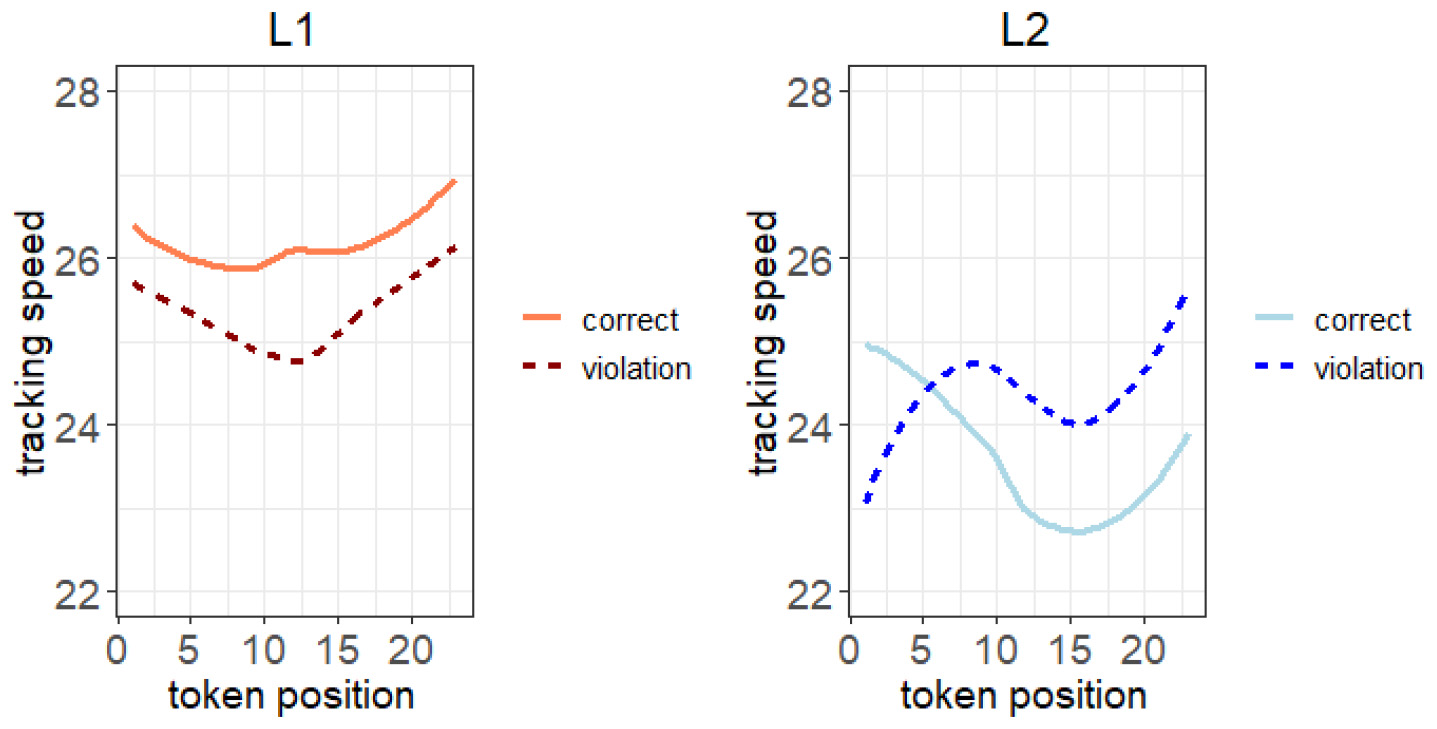
Non-linear regression plots (*ggplot* function, *loess* method) fitting tracking speed of tokens as a function of absolute token position within the stimulus and well-formedness (correct vs. violation), plotted separately for L1 and L2 readers. Since violating tokens occurred at different positions across sentences, curves in the violation condition reflect aggregation over aligned token positions indexed from sentence onset, rather than effects at a single fixed location.

While tracking time provides a straightforward estimate of processing cost, it is inherently influenced by token length, as shown in Figure 1. To better capture moment-to-moment reading efficiency independently of word length, we complemented time-based analyses with a measure of tracking speed. Tracking speed was computed as the ratio between each token length (in number of characters) and its tracking time, yielding a continuous estimate of reading speed as characters per second. This transformation allows processing dynamics to be compared across tokens of different lengths and provides a more direct index of online reading efficiency. Figure 2 shows mean tracking speed as a function of token absolute position for correct and violation sentences, plotted separately for L1 and L2 readers. Since violating tokens occurred at different positions across sentences, position-based effects reflect aggregation over token positions indexed from sentence onset rather than a fixed critical location. Related GAM specifications are provided in Tables A4, A5 in Appendix. Tracking speed profiles revealed distinct temporal dynamics across the two groups, with stronger modulation by sentence well-formedness in L1 readers than in L2 readers. Descriptive statistics further indicated a reduction in median tracking speed for L1 readers (22.97 vs. 21.85 char/s for correct and violation stimuli, respectively), which was statistically significant (*Mann-Whitney* *W* = 6.18*x*10^6^, *p* < 0.001). By contrast, L2 readers showed highly similar median tracking speeds across conditions (18.27 vs. 18.47 char/s), with no significant difference (*Mann-Whitney* *W* = 5.66*x*10^6^, *p* = 0.65).

Thus, L1 readers exhibited a generally higher tracking speed across stimuli, with a clear overall reduction in speed for violation sentences relative to correct ones. In violation sentences, the speed profile for L1 readers displayed a characteristic *V-shaped* pattern, with a transient slowdown followed by a speed recovery toward the end of the stimulus. This pattern is consistent with a temporally circumscribed disruption in processing associated with the presence of a linguistic violation, potentially reflecting predictive and globally coordinated reading mechanisms.

In contrast, L2 readers showed overall slower tracking speeds and a markedly different speed profile as compared to L1 readers. Rather than exhibiting a coherent, focal modulation, their tracking speed displayed more distributed and variable fluctuations across the stimuli. Although the smoothed trajectories for correct and violation sentences appeared to diverge in the initial portion of the stimulus, they subsequently evolved in a largely parallel manner. Given that inferential analyses did not reveal a reliable effect of sentence well-formedness in L2 readers, these descriptive differences should be interpreted cautiously. Overall, the L2 profiles suggest greater variability in tracking dynamics across sentence positions, without clear evidence for robust modulation by linguistic violations.

Since the analysis reported in Figure 2 provides a global description of tracking dynamics across sentence positions, to further disentangle local vs. global effects of linguistic violations, we conducted an additional analysis restricted to stimuli containing a violation. Within these ones, tracking speed at the violated token was contrasted with tracking speed at semantically/grammatically correct tokens. This approach allows us to assess whether processing costs are tightly localized at the anomalous input or instead distributed across the sentence. Correct tokens were defined as all tokens in the same sentence excluding the anomalous token. Importantly, effects reflect aggregation across tokens differing in their position relative to sentence onset.

**Figure 3:**
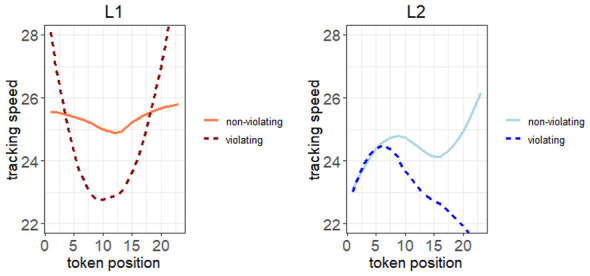
Non-linear regression plots (*ggplot* function, *loess* method) fitting tracking speed of tokens as a function of absolute token position in stimuli containing violations and token status (violating vs. non-violating), plotted separately for L1 and L2 readers. Since violating tokens occurred at different positions, effects reflect aggregation over variable token locations within the stimulus, rather than a single fixed position.

As shown in Figure 3, L1 readers exhibited a pronounced slowdown specifically at the violating token, producing a marked “U-shaped” profile, whereas non-violating tokens displayed only a shallow “V-shaped” decrease in speed. Importantly, this effect is observed despite the fact that violating tokens were distributed across sentence positions (range = 1–23). Nevertheless, the minimum tracking speed consistently occurred around the median violation position across stimuli (median = 10, *M* = 10.43, *SD* = 6.15), suggesting that the observed pattern cannot be attributed to a fixed positional confound. In contrast, L2 readers showed a more variable pattern, with tokens carrying the violation that show a progressively decreasing speed toward the end of the stimuli. This suggests that non-native readers integrate the violation more gradually, relying less on anticipatory mechanisms and more on cumulative, *post-hoc* processing across the sentence.

Overall, these evidence highlight a qualitative difference in the reading strategies of L1 and L2 participants. Native readers show a selective, token-specific slowdown when encountering violations, while non-native readers exhibit a slower, more distributed adjustment, consistent with delayed integrative processing.

### 3.3 Effects of morphosyntactic vs. semantic violations

We next examined whether tracking speed differed as a function of violation type (morphosyntactic vs. semantic) within each group. For L1 readers, no significant difference was observed between morphosyntactic and semantic violations (Kruskal–Wallis χ^2^ = 2.43, *p* = 0.12), indicating that native readers slow down similarly in response to both types of anomalies. In contrast, L2 readers exhibited a significant difference (Kruskal–Wallis χ^2^ = 4.64, *p* = 0.031), with greater slowdown for morphosyntactic than for semantic violations, suggesting that non-native readers are particularly sensitive to formal grammatical discrepancies.

We then compared L1 and L2 readers separately for each violation type. For morphosyntactic violations, L1 readers were substantially faster than L2 readers (χ^2^ = 67.41, *p* < 2.2*e*−16), whereas for semantic violations, L1 readers also outperformed L2 readers, although the group difference was smaller (χ^2^ = 18.09, *p* = < 2.2*e*−5).

Additionally, *post-hoc Dunn* tests (with *Holm* correction) conducted separately for L1 and L2 readers revealed markedly different patterns on morpho-syntactic violations. While L1 readers showed selective slowdowns limited to highly marked constructions, such as Saxon genitives (e.g., *the teacher's book*, see Rosenbach, 2005) and, to a lesser extent, double negation—a construction that may increase processing cost due to its sensitivity to contextual predictability and increased integration demands (Rück et al., 2021)—L2 readers exhibited a redistribution of processing costs across violation subtypes, with even canonical structures such as *SVO* word order (Subject-Verb-Object; e.g., *The boy eats the apple*) eliciting substantial slowdowns. Full results of the *post-hoc* comparisons are reported in Table A6 in Appendix.

Together these findings suggest that L2 readers are generally slower and that this lag is especially pronounced for morphosyntactic violations, consistent with a more reactive, locally driven processing strategy. More generally, these results suggest a more distributed processing cost for morphosyntactic violations in L2 readers, whereas semantic violations tend to elicit more circumscribed effects. Overall, these findings are consistent with a more selective and predictive reading strategy in L1 readers, and a more reactive and distributed processing profile in L2 readers.

### 3.4 Within-word temporal dynamics

To further characterize L1-L2 differences in real-time processing, we complemented the token-level analyses with the temporal dynamics of tracking speed within words. By analyzing successive segments of each token, this approach provides a fine-grained view of how reading behavior unfolds in response to linguistic violations over the course of a word. A within-word perspective is, in fact, particularly informative for distinguishing predictive from reactive processing, as early modulation of tracking speed is expected when violations are anticipated or immediately recognized, whereas delayed effects point to more incremental, locally driven integration.

Overall, L2 readers were significantly slower than L1 readers even at the symbol level (Kruskal–Wallis χ^2^ = 278.66, *p* < 2.2*e*−16), confirming that group differences in reading speed are present at both token- and symbol-level resolutions. When considering all symbols in word token, tracking speed differed reliably between symbols belonging to correct vs. violation stimuli in L1 readers (χ^2^ = 64.08, *p* < 0.001), indicating a robust global effect of sentence-level violations. The same contrast did not reach significance in L2 readers (χ^2^ = 3.38, *p* = 0.066).

**Figure 4:**
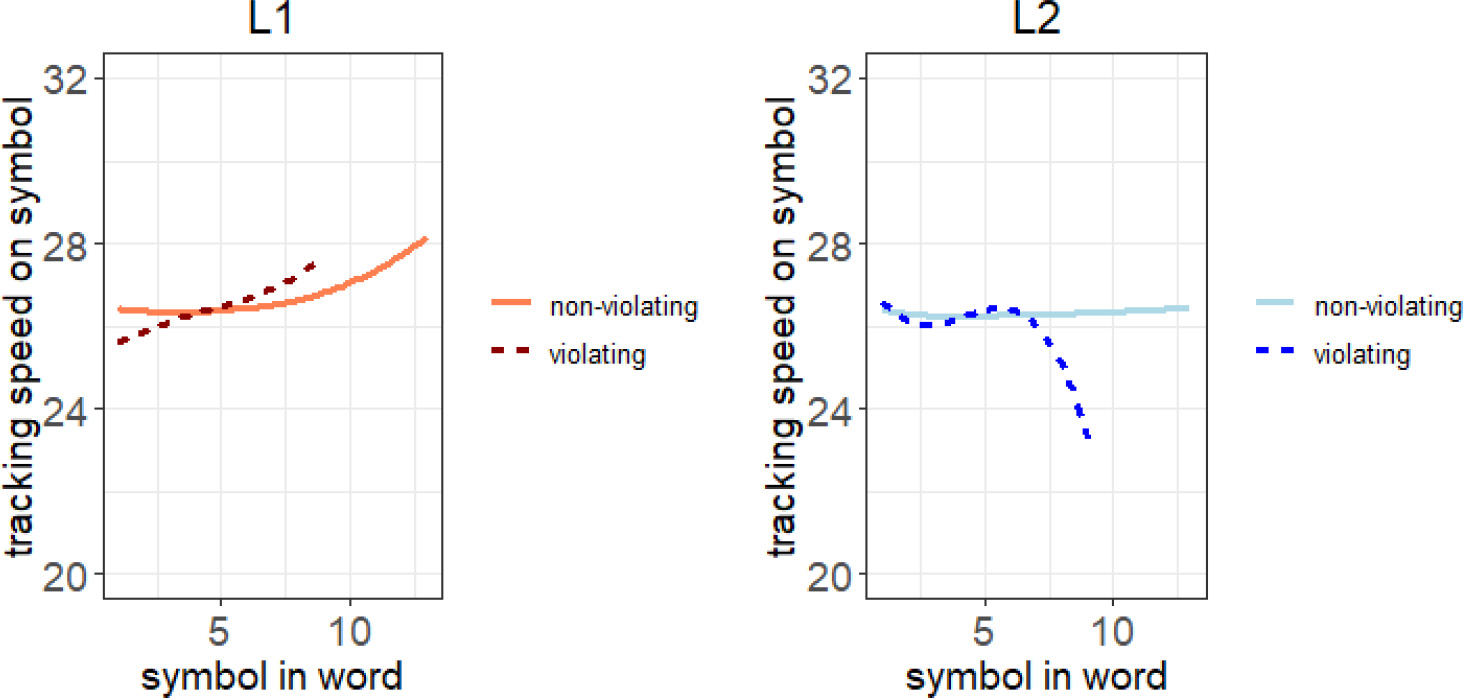
Non-linear regression plots (*ggplot* function, *loess* method) fitting tracking speed on symbol as a function of position in word in stimuli containing violations and token status (violating vs. non-violating), plotted separately for L1 and L2 readers.

We then restricted the analysis to stimuli containing a violation and contrasted symbols belonging to the violating token with symbols from non-violating tokens within the same stimulus. For L1 readers, symbols carrying the violation were tracked significantly more slowly than non-violating symbols (χ^2^ = 15.86, *p* < 0.001). No such difference was observed in L2 readers (χ^2^ = 0.61, *p* = 0.44), with any local divergence being restricted to word-final segments of relatively long words (exceeding mean word length of 4 characters, median 4.14). To visually characterize these effects, Figure 4 shows non-linear regression plots of tracking speed across symbol positions within words, separately for symbols belonging to violating vs. non-violating tokens, in sentences containing a violation. Related GAM specifications are provided in Tables A7, A8 in Appendix.

Together, these results indicate that in L1 readers sensitivity to violations operates both globally across the sentence and locally at the level of the violating symbol, whereas in L2 readers it emerged only at later positions within longer words, suggesting a more gradual and incremental integration process.

### 3.5 Summary of main results

Across analyses, finger-tracking measures reliably differentiated L1 and L2 reading behavior. Considering sentence-wide dynamics derived from token-level analyses, L1 readers showed systematic modulations of tracking speed in response to violations, with violating tokens eliciting sharper and localized slowdowns, whereas L2 readers SSexhibited more variable and less systematic profiles. At the symbol level, violating tokens showed early slowdown in L1 but delayed effects in L2.

Together, these results indicate both quantitative and qualitative differences in the temporal dynamics of reading between native and non-native speakers, observable across token- and symbol-level measures.

Overall, the present analyses show that (i) finger-tracking yields systematic and group-sensitive measures of reading speed, (ii) these measures are modulated by the presence and type of linguistic violations, and (iii) they reveal distinct temporal profiles across groups in the unfolding of sentence processing.

## 4 Discussion

### 4.1 General overview of findings

Our results provide novel insights into the online dynamics of sentence processing in L1 and L2 readers, as captured through fine-grained finger-tracking measures.

Across analyses, finger-tracking measures reliably differentiated L1 and L2 reading behavior. Native readers were consistently faster than L2 readers and showed higher comprehension accuracy across conditions, confirming robust group differences in overall reading efficiency.

These global differences were observed across multiple levels of analysis, including sentence-wide dynamics derived from token position, as well as token- and symbol-level measures.

Importantly, the finger-tracking paradigm proved to be sensitive to linguistic well-formedness, capturing both overall slowdowns and fine-grained temporal modulations associated with processing surprisal and difficulty.

### 4.2 Predictive vs. reactive processing

When focusing on individual tokens within violation-containing sentences, the effect of the critical token differed markedly between groups. In L1 readers, the token introducing the violation elicited an immediate and pronounced slowdown, whereas in L2 readers, the effect was more gradual, with tracking speed declining progressively toward the sentence end.

These patterns are consistent with the overall token-level analyses: sensitivity to violations emerged early and robustly in L1 readers, whereas in L2 readers no uniform token-level contrast was observed. This suggests that anomaly-related effects in non-native reading does not operate continuously across the sentence, but rather unfold more gradually across the processing stream.

Collectively, these findings are broadly consistent with previous evidence suggesting that native speakers engage fast, predictive-driven mechanisms during sentence comprehension, including during the processing of syntactic or semantic anomalies (Staub and Goddard, 2019; Rayner, 1998), with neural responses to words modulated by their informational content and effort required to update the reader's mental model (Wang et al., 2026; Frank et al., 2015). Importantly, this literature typically does not involve continuous, time-resolved tracking of visual-motor behavior. In contrast, evidence from L2 reading suggests more incremental and integration-based processing profiles, which have been described in terms of reduced predictive processing and greater reliance on bottom-up information (Hopp, 2016, 2015b; Clahsen and Felser, 2006).

### 4.3 Morphosyntactic vs. semantic violations

Analyses examining the effect of violation type revealed clear differences between L1 and L2 readers. For L1 readers, no significant difference in tracking speed was observed between morphosyntactic and semantic violations, indicating that native speakers slow down similarly in response to both types of anomalies. This suggests that predictive processing in L1 is engaged continuously, and the detection of a violation—be it morphosyntactic or semantic—elicits a comparable adjustment in reading speed. In contrast, L2 readers exhibited greater sensitivity to morphosyntactic violations than to semantic ones, suggesting that non-native readers are particularly affected by formal structural discrepancies. Morphosyntactic violations may engage more distributed processing demands, whereas semantic violations appear to produce more circumscribed effects.

The pattern observed in L2 readers may reflect a more locally driven and incremental processing profile, in which formal grammatical violations require more effortful processing, whereas native readers rely on continuous predictive monitoring that responds similarly to both types of anomalies, revealing slowdowns limited to highly marked constructions (Hagoort, 2003). Notably, this interpretation should be considered in light of differences in language proficiency between the two groups. Although the L2 participants in the present study represented relatively advanced learners, differences in cumulative language experience and processing automaticity relative to native speakers may still have influenced the efficiency of online linguistic integration.

More generally, this interpretation aligns with evidence from ERP studies showing that the processing of individual words during reading is shaped by interactions between lexical-level information and message-level representations, and that these integrative processes are modulated by reading proficiency (Wang et al., 2026; Hopp, 2016; Stafura and Perfetti, 2014).

### 4.4 Temporal dynamics within words

Analyzing reading at the symbol level within each word provided a more fine-grained perspective on how linguistic violations are detected and processed in real time. This approach allowed us to distinguish between early, predictive adjustments and later, reactive integration, complementing the token-level analysis.

Our symbol-level results revealed clear differences between L1 and L2 readers. In L1 readers, symbols within violating tokens elicited an early slowdown, observable at the word onset. This effect suggests that native readers engage in rapid, anticipatory processing, dynamically adjusting reading speed as soon as a potential violation is encountered. The early emergence of this slowdown aligns with continuous predictive monitoring, consistent with prior evidence from eye-tracking and ERP studies showing rapid detection of syntactic and semantic anomalies (Staub and Goddard, 2019; Rayner, 1998).

In contrast, L2 readers did not show an immediate symbol-level effect for violating tokens. Sensitivity to violations appears to be limited to later positions within the word, particularly in longer tokens. This pattern may be consistent with a more gradual and incremental integration process, whereby anomaly-related effects become observable only after sufficient lexical and syntactic information has been accumulated. Such a pattern is broadly consistent with accounts proposing that non-native readers rely more heavily on reactive, bottom-up mechanisms, rather than continuous prediction (Hopp, 2016, 2015b; Clahsen and Felser, 2006).

Taken together, the symbol-level analyses reinforce and extend the sentence- and token-level findings. They suggest that the temporal unfolding of reading behavior differs both quantitatively and qualitatively between native and non-native readers: while L1 reading is characterized by earlier and more broadly distributed sensitivity to anomalies, L2 reading appears to exhibit more gradual and context-dependent responses to unexpected input.

Crucially, these patterns are compatible with hierarchical accounts of linguistic prediction, which posit that predictive processes operate at multiple representational levels and unfold over different time scales during word recognition (Heilbron et al., 2022). From this perspective, the early within-word slowdowns observed in L1 readers may reflect rapid prediction error signals at sublexical or lexical levels, whereas the delayed effects in L2 readers suggest that predictive mechanisms are either weaker or shifted to later stages of integration.

Our results have important theoretical implications. First, they provide behavioral evidence that predictive mechanisms in L1 operate across multiple levels of granularity, from sentence structure down to individual symbols within words. Second, they highlight that L2 reading, even in upper-intermediate readers, may be constrained by the temporal dynamics of processing, resulting in delayed responsiveness to anomalies.

### 4.5 Methodological and theoretical implications

Our study suggests that finger-tracking provides a continuous, fine-grained behavioral measure for real-time reading dynamics, complementing traditional eye-tracking and ERP measures (Kessler et al., 2021). By capturing dynamic adjustments in tracking speed, this method reveals how readers respond to linguistic anomalies at multiple levels—sentence, token, and symbol.

Differences between L1 and L2 readers suggest that non-native readers may compensate for less efficient predictive mechanisms by relying more on cumulative lexical and syntactic information across the sentence, resulting in slower and more gradual sensitivity to violations. This highlights quantitative and qualitative differences in processing strategies and reinforces the utility of finger-tracking for probing the temporal micro-dynamics of reading.

Notably, the temporal dynamics observed at the symbol level parallel ERP findings, which consistently report earlier neural responses to violations in L1 readers and delayed or attenuated responses in L2 readers (Hopp, 2015b; Clahsen and Felser, 2006; Rayner, 1998). Similar tendencies have also been reported in eye-tracking studies of sentence reading, where L2 readers often show reduced anticipatory processing and greater reliance on later integrative mechanisms relative to native readers (Kim and Grüter, 2021; Staub and Goddard, 2019; Hopp, 2016). This convergence indicates that finger-tracking captures behavioral manifestations of predictive vs. reactive processing, providing a continuous, real-time index that complements established methodologies for investigating online language processing—such as neurophysiological or eye-tracking measures.

From a theoretical perspective, these results support models of sentence processing that emphasize gradient predictability and incremental integration, and are compatible with hierarchical accounts of linguistic prediction in which anticipatory processes operate across multiple temporal and representational scales (Heilbron et al., 2022). Crucially, our findings show that such distinctions are observable not only in neural signals, but also in continuous behavioral dynamics.

From an applied standpoint, understanding how readers adjust reading speed in response to anomalies can inform educational and assessment practices. Distinct temporal profiles may serve as behavioral markers of processing efficiency, helping identify learners who rely more heavily on reactive strategies, and potentially guiding interventions aimed at strengthening anticipatory processing in L2 readers, for example through guided sentence-prediction exercises or structured exposure to language-specific highly constraining contexts (e.g., non-canonical word order, morphosyntactic dependencies, or constructions associated with frequent learner difficulties).

Overall, our findings reinforce the value of finger-tracking as a scalable and sensitive method for investigating real-time reading strategies and comprehension, bridging theoretical models of predictive processing with practical applications in assessment and pedagogy.

Several limitations should be however acknowledged. Our sample size was relatively small, although participant samples of this scale are not uncommon in controlled psycholinguistic studies involving fine-grained behavioral measures of L2 processing (e.g., Soares et al., 2019). In addition, L2 proficiency was assessed via self-report rather than objective measures, which may constrain the interpretation of group differences. Future studies should examine whether these patterns hold with larger samples and broader proficiency ranges, and across different types of linguistic anomalies, to establish the robustness and generalizability of the present findings.

## Data Availability

Text-aligned data supporting the conclusions of this article will be made available by the authors, without undue reservation.
