# Supplementary material for: Finger-tracking captures distinct cognitive signatures in L1 vs. L2 reading
Source: Front Psychol. 2026 Jul 1;17:1792437. doi: 10.3389/fpsyg.2026.1792437 (PMC13369239; doi:10.3389/fpsyg.2026.1792437)
Supplement: Supplementary file 1 [file Data_Sheet_1.pdf]

Supplementary Material

1 EXPERIMENTAL STIMULI

The full set of experimental stimuli used in the present study is provided below. Stimuli containing linguistic violations are marked with an asterisk (*), whereas the specific violating token is indicated in boldface for clarity of presentation.

These annotations were not present during the experimental procedure and are included solely to facilitate transparency and reproducibility of stimulus construction.

The stimulus set was divided into two counterbalanced lists. Each list contained 8 well-formed sentences and 8 sentences with linguistic violations. Participants were assigned to one of the two stimulus lists in a counterbalanced manner within each language group (L1 and L2), such that half of the participants in each group completed List 1 and the remaining half completed List 2. Each participant saw only one version of each stimulus item. Violations targeted both (morpho-)syntactic and semantic levels, with the aim of eliciting general predictive disruption rather than effects tied to a single violation type. Morphosyntactic violations included phrasal verb errors, subject-verb agreement violations, word order (SVO) deviations, passive constructions, and double negation, whereas semantic violations comprised lexical selection anomalies and idiomatic substitutions.

List of stimuli 1

Spring break was close. Finally, the day came: Margaret got through the exam without problems and left for Cuba light-hearted.

* Mike was late, his wife and children were already waiting for him at home, so he got **down from** the car quickly and went inside the store to buy a couple of things for dinner.

“Can you please turn the volume down? It’s the 100th time I’m asking you!” yelled Sandy’s mother while she was watching her favourite TV series on the sofa.

Lucy noticed Sarah’s book was on the table because its cover was really beautiful and elegant.

The door of the house was open, so the cat managed to run inside to hide from the rain, brought by a mid-summer storm.

*The professor asked Sophie a question: **the answer she gave** quickly, her voice steady but her eyes betraying the uncertainty she felt.

He spoke fluently several languages, impressing everyone at the conference with his knowledge and ease of communication.

*After a long day of travel, **into the room** he slowly walked, his tired feet dragging with each step, his eyes were heavy, and his body longed for nothing more than sleep.

The old house was broken into while the owners were on vacation in the mountains, and the thieves stole valuable antiques that had been passed down through generations

*The car is **repairing** at the local garage and it should be ready by the end of the week, if all goes as planned.

*I can’t go **nowhere** this weekend because I have a ton of work to finish, and I promised I would help my friend move into her new apartment.

*I don’t have **none** of the answers to the questions on the test, but I plan to study hard tonight and ask the teacher for some clarification tomorrow.

The volcano was meticulously observed by a team of experienced scientists using advanced thermal imaging equipment to monitor its subtle geological changes.

*The **sofa** was peacefully sleeping on the cat, when a glass suddenly fell from the kitchen counter hitting the floor and shattering in hundred pieces.

I just had to persuade him a little to tell me the truth, and he quickly spilled the beans, admitting that he was the one who ate the last cookie.

*Tom was feeling a bit under the **bridge**, so after the game, instead of going out to celebrate the victory with his friends, he went back home.

List of stimuli 2

* Spring break was close. Finally, the day came: Margaret got through **of** the exam without problems and left for Cuba light-hearted.

Mike was late, his wife and children were already waiting for him at home, so he got out of the car quickly and went inside the store to buy a couple things for dinner.

* “Can you please turn the volume **of**? It’s the 100th time I’m asking you!” yelled Sandy’s mother while she was watching her favourite TV series on the sofa.

* Lucy noticed the book **of Sarah** was on the table, because its cover was really beautiful and elegant.

*The **house’s** door was open, so the cat managed to run inside to hide from the rain, brought by a mid-summer storm.

The professor asked Sophie a question: she gave the answer quickly, her voice steady but her eyes betraying the uncertainty she felt.

***Spoke he** fluently several languages, impressing everyone at the conference with his knowledge and ease of communication.

After a long day of travel, he slowly walked into the room, his tired feet dragging with each step, his eyes were heavy, and his body longed for nothing more than sleep.

*The old house **has** broken into while the owners were on vacation in the mountains, and the thieves stole valuable antiques that had been passed down through generations.

The car is being repaired at the local garage and it should be ready by the end of the week, if all goes as planned.

I can’t go anywhere this weekend because I have a ton of work to finish, and I promised I would help my friend move into her new apartment.

I don’t have any of the answers to the questions on the test, but I plan to study hard tonight and ask the teacher for some clarification tomorrow.

*The volcano was meticulously **eaten** by a team of experienced scientists using advanced thermal imaging equipment to monitor its subtle geological changes.

The cat was peacefully sleeping on the sofa, when a glass suddenly fell from the kitchen counter hitting the floor and shattering in hundred pieces.

*I just had to persuade him a little to tell me the truth, and he quickly spilled the **milk**, admitting that he was the one who ate the last cookie.

Tom was feeling a bit under the weather, so after the game, instead of going out to celebrate the victory with his friends, he went back home.
