# Supplementary material for: Finger-tracking captures distinct cognitive signatures in L1 vs. L2 reading
Source: Front Psychol. 2026 Jul 1;17:1792437. doi: 10.3389/fpsyg.2026.1792437 (PMC13369239; doi:10.3389/fpsyg.2026.1792437)
Supplement: Supplementary file 2 [file Supplementary_file_1.pdf]

frontiers

Supplementary Material

DETAILED MODEL RESULTS

To complement the descriptive analyses and visualisations presented in the main text, we fitted a series of Generalised Additive Models (GAMs). These models provide a formal assessment of the global temporal trajectories in tracking time and speed across the different levels of analysis, capturing non-linear trends and overall group differences. While fine-grained local modulations are primarily illustrated through the descriptive plots discussed in the main text, the GAM results can be interpreted as complementary evidence for the robustness of the observed temporal patterns.

Table S1. Summary of a GAM fitting tracking time across groups as a function of word length. Reference level: L1.

Term	Estimate / edf	SE / Ref.df	t / F	p-value
intercept (L1)	0.03	0.02	1.32	> 0.05
intercept (L2)	0.01	0.03	0.26	> 0.05
length (L1)	0.05	0.01	61.74	$< 2e - 16$
length (L2)	0.01	0.001	10.24	$< 2e - 16$
participants (random)	29.76	30	127.9	$< 2e - 16$
adjusted $R^2 = 0.498$ — Deviance explained = 49.9%				

Table S2. Summary of a GAM fitting tracking time across groups as a function of word frequency (log-transformed). Reference level: L1.

Term	Estimate / edf	SE / Ref.df	t / F	p-value
intercept (L1)	0.56	0.02	24.50	$< 2e - 16$
intercept (L2)	0.13	0.03	4.16	$< 4e - 05$
(log) frequency (L1)	-0.03	0.001	-43.97	$< 2e - 16$
(log) frequency (L2)	-0.01	0.001	-7.49	$< 8e - 14$
participants (random)	29.73	30	110.3	$< 2 \times 10^{-16}$
adjusted $R^2 = 0.406$ — Deviance explained = 40.8%				

Table S3. Summary of a GAM fitting tracking time across groups as a function of word-length groups (short vs. long tokens). Reference levels: L1, short tokens.

Term	Estimate / edf	SE / Ref.df	t / F	p-value
intercept (L1, short tokens)	0.15	0.02	7.83	$< 6e - 15$
intercept (L2, short tokens)	0.04	0.03	1.33	> 0.05
long tokens (L1)	0.20	0.004	48.91	$< 2e - 16$
long tokens (L2)	0.05	0.005	8.08	$< 7e - 16$
participants (random)	29.69	30	106.2	$< 2 \times 10^{-16}$
adjusted $R^2 = 0.408$ — Deviance explained = 41.0%				

Table S4. Summary of a GAM fitting tracking speed for L1 as a function of token position for well-formedness (correct vs. violation). Reference level: correct stimuli.

Term	Estimate / edf	SE / Ref.df	t / F	p-value
intercept (correct)	25.99	1.346	17.85	$< 2e - 16$
intercept (violation)	-0.75	0.62	-1.20	> 0.05
token position (correct)	-0.01	0.04	-0.17	> 0.05
token position (violation)	0.02	0.06	0.32	> 0.05
participants (random)	14.78	15	68.33	$< 2 \times 10^{-16}$
	adjusted $R^2 = 0.13$	—	Deviance explained = 13.2%	

Table S5. Summary of a GAM fitting tracking speed for L2 as a function of token position for well-formedness (correct vs. violation). Reference level: correct stimuli.

Term	Estimate / edf	SE / Ref.df	t / F	p-value
intercept (correct)	25.68	2.64	9.74	$< 2e - 16$
intercept (violation)	-2.45	0.73	-3.38	< 0.001
token position (correct)	-0.13	0.05	-2.62	< 0.01
token position (violation)	0.17	0.07	2.49	< 0.05
participants (random)	14.92	15	172.6	$< 2 \times 10^{-16}$
	adjusted $R^2 = 0.278$	—	Deviance explained = 28.0%	

Table S6. *Dunn* multiple comparison, with *Holm* adjusted p-values, on morpho-syntactic subtype violations.

Comparison	Z	adj. p-value
L1		
double negation – passive	3.13	< 0.05
double negation – phrasal verb	1.00	> 0.05
passive – phrasal verb	-2.41	> 0.05
double negation – Sax genitive	5.33	< 0.001
passive – Sax genitive	2.37	> 0.05
phrasal verb – Sax genitive	4.80	< 0.001
double negation – SVO	2.21	> 0.05
passive – SVO	-1.19	> 0.05
phrasal verb – SVO	1.34	> 0.05
Sax genitive – SVO	-3.65	< 0.01
L2		
double negation – passive	10.59	< 0.001
double negation – phrasal verb	9.84	< 0.001
passive – phrasal verb	-1.83	> 0.05
double negation – Sax genitive	13.28	< 0.001
passive – Sax genitive	3.44	< 0.01
phrasal verb – Sax genitive	5.40	< 0.001
double negation – SVO	8.42	< 0.001
passive – SVO	-3.13	< 0.01
phrasal verb – SVO	-1.48	> 0.05
Sax genitive – SVO	-6.57	< 0.001

Table S7. Summary of a GAM fitting symbol tracking speed for L1 as a function of token position in stimuli containing violations and token status (violating vs. non-violating). Reference level: non-violating tokens.

Term	Estimate / edf	SE / Ref.df	t / F	p-value
intercept (non-violating)	25.46	1.45	17.56	$< 2e - 16$
intercept (violating)	0.93	0.92	1.01	> 0.05
token position (non-violating)	0.27	0.06	4.51	< 0.001
token position (violating)	-0.88	0.27	-3.28	$= 0.001$
participants (random)	14.89	15	135.8	$< 2 \times 10^{-16}$
adjusted $R^2 = 0.13$ — Deviance explained = 13.2%				

Table S8. Summary of a GAM fitting symbol tracking speed for L2 as a function of token position in stimuli containing violations and token status (violating vs. non-violating). Reference level: non-violating tokens.

Term	Estimate / edf	SE / Ref.df	t / F	p-value
intercept (non-violating)	22.73	2.34	9.73	$< 2e - 16$
intercept (violating)	0.30	0.91	0.33	> 0.05
token position (non-violating)	0.36	0.06	5.97	< 0.001
token position (violating)	-0.24	0.27	-0.90	> 0.05
participants (random)	14.96	15	349.3	$< 2 \times 10^{-16}$
adjusted $R^2 = 0.28$ — Deviance explained = 28.4%				
